# Understanding Surface Reaction of Acrylosilane–Melamine Clearcoat with Phenyl Isocyanate

**DOI:** 10.1021/acs.langmuir.6c02112

**Published:** 2026-08-06

**Authors:** Yiwen Guo, Jihyeong Ryu, Juseok Choi, Sibing Chen, Xing Chen, Carl Brent Douglas, Delson Trindade, Yongqing Huang, Yun Jing, Padraic O’Reilly, Sung Park, Seong H. Kim

**Affiliations:** † Department of Chemical Engineering and Materials Research Institute, 8082 Pennsylvania State University, University Park, Pennsylvania 16802, United States; ‡ Institute of Molecular Plus, School of Chemical Engineering and Technology, Tianjin University, Tianjin 300192, P. R. China; § Global Innovation Center, Axalta Coating Systems, Philadelphia, Pennsylvania 19112, United States; ∥ Molecular Vista, 6840 Via del Oro, Suite 110, San Jose, California 95119, United States

## Abstract

Urethane adhesives are critical to the structural integrity and long-term durability of automotive glazing systems, yet robust bonding of the windshield to chemically inert, cross-linked clearcoat surfaces of the vehicle body remains a persistent challenge. This study systematically investigates the molecular-level mechanisms governing the reactivity of automotive clearcoat surfaces toward isocyanate-based adhesives, focusing on the effects of aqueous pH pretreatment and solvent swelling. A model acrylosilane–melamine clearcoat was subjected to controlled hydrolysis under acidic, neutral, and basic conditions to modulate surface silanol (Si–OH) density. Phenyl isocyanate (Ph-NCO), chosen for its distinct aromatic spectroscopic signature, was employed as a model isocyanate to probe interfacial reactivity, forming the urethane bond to the clearcoat surface, both in vapor phase and in *N*-methyl-2-pyrrolidone (NMP) solution. Photoinduced force microscopy (PiFM) was used to probe subsurface structural changes, and vibrational sum-frequency generation (SFG) spectroscopy was used to probe the topmost surface chemistry. From these analyses, it was found that acidic and basic pretreatments significantly increase the density of reactive OH groups exposed to the topmost surface, leading to enhanced urethane formation upon exposure to isocyanate vapor. In the solution-phase reaction, the NMP solvent induces swelling and reorganization of polymer chains in the surface region, thus allowing reactions with subsurface OH groups. These findings elucidate molecular-level factors governing the reactivity of clearcoat OH groups toward isocyanates and provide mechanistic insight into interfacial reactions relevant to urethane adhesive bonding.

## Graphical abstract

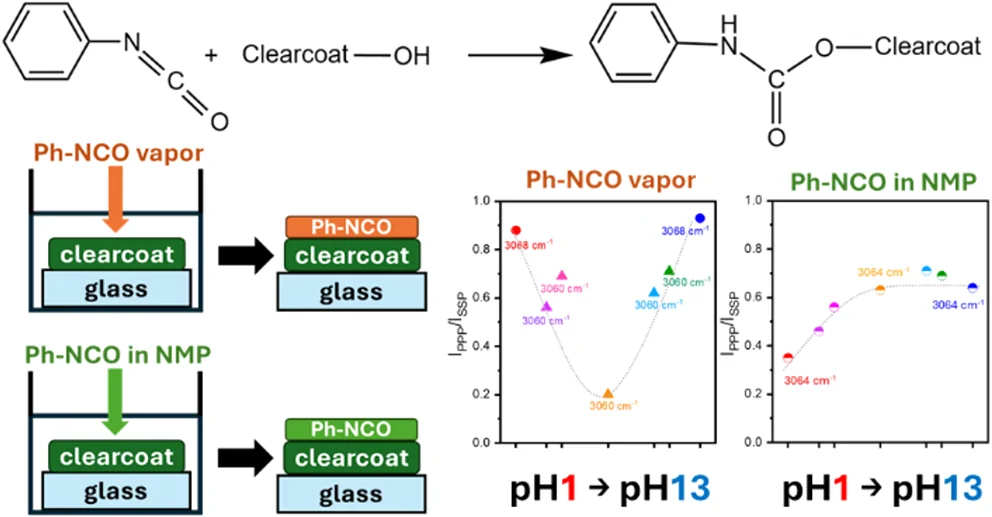

## Introduction

Urethane adhesives play an important role in modern automotive manufacturing, particularly for bonding windshields to the vehicle body, where mechanical integrity, safety, and long-term durability are paramount. By replacing earlier mechanical fastening methods, these adhesives enable the windshield to function as a structural component, significantly enhancing the torsional rigidity and crash resistance of the vehicle.^1,2^ Commercially available systems are predominantly one-component, moisture-curing polyurethane adhesives.^3^ These formulations are based on isocyanate-terminated prepolymers, which react with ambient water to form cross-linked polyurea networks. This curing mechanism imparts excellent mechanical resilience, including high tensile strength and toughness—properties essential for accommodating dynamic loading, thermal expansion, and vibration across a wide range of environmental conditions.^3,4^

The efficacy of these urethane adhesives is strongly governed by the nature of interfacial bonding to both glass and painted vehicle substrates. While glass surfaces are often treated with silane-based primers to enhance adhesion, bonding to the coating surfaces of the vehicle body is more complex due to the chemically inert nature of automotive clearcoats.^5–11^ These topmost layers are commonly composed of cross-linked acrylosilane–melamine networks (Scheme 1), which are produced from a tailored mixture of acrylic polyols, carbamate oligomers, alkylated melamine cross-linkers, and acrylosilane components bearing methoxysilyl (−Si–OCH_3_) functionalities.^12^ This clearcoat system renders gloss, UV resistance, and environmental durability to the underlying colorcoat layers.^2,13^ However, these same properties contribute to low surface energy and limited chemical reactivity, complicating efforts to achieve robust adhesion.^12,14^

**Scheme 1:**
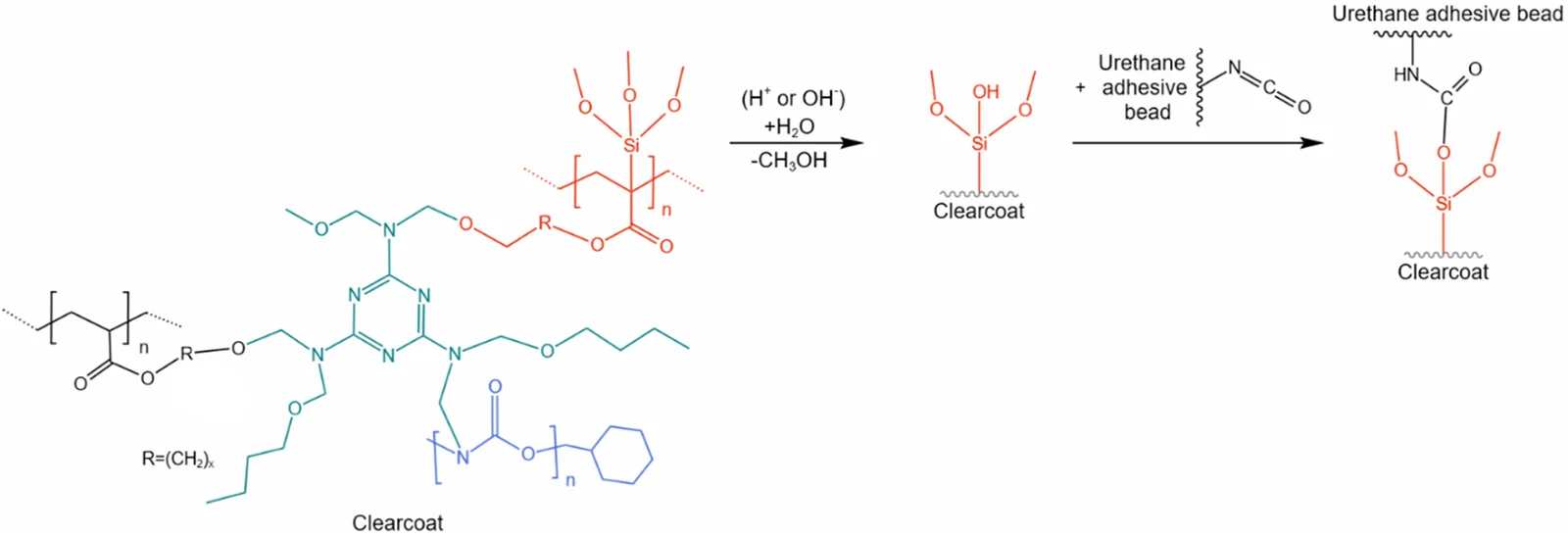
Chemical Structure and Interfacial Bonding Mechanism between the Acrylosilane–Melamine Clearcoat and Urethane Adhesive^a^

a In the clearcoat structure, the red unit is acrylosilane with methoxysilyl groups that hydrolyze to silanol groups; the green unit is melamine-formaldehyde crosslinker; the blue unit is carbamate oligomer; the black unit in the clearcoat is an acrylic polyol segment.^12^

The central hypothesis involved in urethane adhesive bonding to the clearcoat surface is that the hydrolysis of surface-exposed methoxysilyl groups produces silanol (−Si–OH) groups, which act as reactive anchoring sites for covalent bonding with isocyanate (−NCO) functionalities present in urethane adhesives.^6,12^ As illustrated in Scheme 1, these silanols can undergo nucleophilic addition with isocyanates to form urethane linkages, thus establishing a chemically bonded interface between the clearcoat and the urethane adhesive. This interfacial chemistry is essential for achieving durable bonding performance in automotive glass mounting processes.^3,4^

However, directly analyzing the buried interface formed after applying a commercial urethane adhesive presents a significant challenge. These commercial adhesives typically comprise a complex mixture of urethane polymers, carbon black, limestone, solvent, and various organic additives.^15^ The resulting adhesive bead is chemically heterogeneous, optically opaque, mechanically thick, and physically sticky—properties that severely limit the use of conventional surface-sensitive spectroscopic and microscopic techniques. These complications hinder direct molecular-level analysis of the clearcoat–adhesive interface, making it difficult to isolate the contributions of specific chemical interactions responsible for adhesion. Although peel-off and lap-shear tests are standard methods for validating adhesion strength in industrial settings, such empirical methods do not provide any information relevant to molecular-level mechanisms that govern interfacial bonding.^3,16^

To circumvent these experimental limitations, this study employs phenyl isocyanate (Ph-NCO) as a model isocyanate species to systematically probe the interfacial reactivity of clearcoat surfaces. Phenyl isocyanate offers several advantages for surface analysis; it contains the isocyanate group capable of forming urethane linkages, and its aromatic structure provides spectroscopically distinct signatures.^17^ The acrylosilane–melamine clearcoat surface was pretreated with acidic, neutral, and basic solutions as a means to convert the surface-exposed methoxysilyl groups to hydroxyl groups (Scheme 1).^5–11^ In parallel, the role of *N*-methyl-2-pyrrolidone (NMP), a common solvent in commercial formulations, is investigated to assess its ability to expose buried reactive groups within the clearcoat. Photoinduced force infrared (PiF-IR) spectroscopy and vibrational sum-frequency generation (SFG) spectroscopy were employed for interfacial characterization. PiF-IR was able to differentiate chemical changes in the subsurface regions of the clearcoat after the pretreatment. By detecting signals from the phenyl groups of the reaction product remaining at the topmost surface, SFG enabled direct comparison of reaction extents across various treatment conditions.^18–21^ Together, these investigations explain how surface hydrolysis and solvent swelling modulate the chemical reactivity of clearcoat surfaces toward isocyanates. The resulting insights will inform the rational design of surface preparation protocols and adhesive formulations for automotive glazing systems.^22^

Our previous study established how pH treatment and NMP exposure modify the surface chemistry of acrylosilane–melamine clearcoats through hydrolysis and solvent-induced swelling. The present work focuses on a subsequent question: whether the resulting surface and subsurface OH groups are accessible and reactive toward isocyanates. Using Ph-NCO as a model isocyanate probe molecule, this study investigates urethane-forming reactions and interfacial structure at the clearcoat surface. Because Ph-NCO is employed as a simplified model system, the results should be interpreted as a mechanistic insight into interfacial reactivity rather than a direct evaluation of commercial adhesive performance.

## Experimental Methods

In this work, a model clearcoat formulation was employed that closely resembled the composition of a commercial one-component (1K) acrylosilane–melamine clearcoat. This material was supplied by Axalta Coating Systems, Ltd.^12^ NMP (purity = 99.5%) was purchased from Thermo Scientific. 1,1,1,3,3,3-Hexamethyldisilazane (HMDS) (purity = 98%) was purchased from Acros Organics. Phenyl isocyanate (purity ≥ 98%) was purchased from Sigma-Aldrich. Diiodomethane (CH_2_I_2_) (purity = 99%) was purchased from Sigma-Aldrich.

Clearcoat films were prepared by applying a premixed precursor solution onto glass slides using the drawdown technique, followed by thermal curing in a box furnace.^12^ The curing was done by ramping the temperature at 5 °C/min to 140 °C, holding for 30 min, and then cooling to room temperature at a rate of 1 °C/min. Differential scanning calorimetry analysis indicated a glass transition temperature of ∼60 °C for the fully cured clearcoat.^12^

All experiments were conducted at room temperature. Clearcoat surfaces were pretreated with aqueous solutions at pH 1, 7, or 13 for 10 min, rinsed with deionized water, and dried under nitrogen. For a vapor-phase exposure, Ph-NCO vapor was generated by bubbling dry nitrogen through a liquid Ph-NCO reservoir and directing this stream onto the pretreated surfaces for 10 min in a hood. The saturated vapor pressure of Ph-NCO was estimated to be 0.2 kPa at room temperature.^23^ The pretreated clearcoat samples were also exposed to a solution of Ph-NCO in NMP at a 1:20 volume ratio for 10 min, followed by rinsing with NMP and drying by blowing with nitrogen. Control samples without isocyanate exposure were also prepared. Clean fused quartz (water contact angle <10°) and hexamethyldisilazane (HMDS)-modified fused quartz (water contact angle ∼75°) were included as control references for testing the surface reaction of the silanol group with Ph-NCO vapor and in a Ph-NCO/NMP solution.

Contact angle measurements of the clearcoat surfaces pretreated with aqueous solutions with different pHs and reacted with Ph-NCO were performed using a Ramé-Hart Model 260 automated goniometer. Surface energy was calculated based on the contact angles of diiodomethane and water using Fowkes theory.^24^ The dispersive and polar components of surface energy are 50.8 and 0 mJ/m^2^ for CH_2_I_2_, and 26.4 and 46.4 mJ/m^2^ for H_2_O, respectively.

Photoinduced force microscopy (PiFM) was conducted with a Vista 200 microscope (Molecular Vista, CA) equipped with a PT277-XIR optical parametric oscillator (Ekspla, Lithuania), with a tuning range of 554–2050 and 2250–4000 cm^–1^ and a wavenumber resolution of <5 cm^–1^. The IR laser beam (pulse width = 8 ps; *p*-polarized) was focused on the tip apex of an atomic force microscopy probe (NCH-PtIr probe from Nanosensors, nominal resonance frequency = 300 kHz) via a parabolic mirror with a focal length of 3 mm, producing a laser spot size of λ × 1.5λ (where λ is the IR wavelength) on the sample surface. The average power of the focused beam was 0.1–0.5 mW. The spectral analysis was conducted in two modes: (i) surface-sensitive mode through a sideband modulation (i.e., heterodyne detection with the pulse modulation frequency of 1.6 MHz) and (ii) bulk-sensitive mode via a direct-drive modulation (i.e., homodyne detection while keeping the pulse modulation frequency locked at the first resonance with the tip engaged via feedback on the second mechanical resonance).^25,26^ The estimated probe depth was around 20 nm for the surface-sensitive mode and on the order of 1 μm for the bulk-sensitive mode. Each sample was imaged with PiFM over an area of 1 × 2 μm^2^ with a resolution of 256 × 256 pixels; then PiF-IR spectra over a full spectral range were recorded at 6 locations with a sweep time of 15 s. All PiFM and PiF-IR data analysis were carried out using SurfaceWorks.

Vibrational sum-frequency generation (SFG) spectroscopy was performed using a scanning picosecond SFG spectrometer (Ekspla, Lithuania). A detailed description of the system is provided in a previous study.^27^ The SFG signal was generated by overlapping a visible laser pulse (532 nm; repetition rate = 10 Hz) with a tunable IR pulse from an optical parametric generator and amplifier, both in space and time. Measurements of SFG signals were conducted using *ssp* and *ppp* polarization combinations, where the three sequential *s* and *p* letters represent the polarization states of the SFG signal, visible beam, and IR beam, respectively. The visible and IR beams were incident at angles of 60 and 56°, respectively, with respect to the surface normal. The SFG spectra were collected with a 4 cm^–1^ increment of the incident IR beam over the 2800–3100 cm^–1^ range and a 10 cm^–1^ increment over the 3100–3800 cm^–1^ range. Each data point in a single measurement was averaged over 300 laser shots. The resulting SFG intensities were normalized to the input powers of the IR and visible beams.

To assist the SFG spectral interpretation, the normal modes of phenyl isocyanate and phenyl urethane were theoretically predicted using the NWChem software package.^28^ In the case of urethane, the surface atom to which the oxygen of the carbamate group is bonded is modeled with an iodine atom that does not couple vibrationally due to a large atomic mass difference from other constituent atoms.^17,29,30^ Although iodine and silicon differ in electronegativity and bonding character, the iodine-capped model is used here primarily to minimize vibrational coupling with the terminal capping atom rather than to fully reproduce the structure of an extended silicon-containing surface. Therefore, small shifts in the absolute frequencies of modes localized near the urethane-surface linkage cannot be excluded; however, this approximation is not expected to alter the phenyl-ring C–H mode assignments or the qualitative dipole/polarizability trends used for SFG interpretation.^29,30^ Geometry optimizations and vibrational frequency analyses were performed within the framework of density functional theory (DFT), employing the B3LYP functional with the 6–311++G­(d,p) basis set. Default convergence thresholds for maximum and root-mean-square forces and displacements were used to ensure structural accuracy and stability. Phenyl isocyanate was determined to exhibit only a single stable conformation, while multiple conformers were identified for phenyl urethane. Based on energy evaluation and structural stability analysis, the lowest-energy conformation of phenyl urethane was selected for further computations. The vibrational modes were calculated under the harmonic oscillation approximation. To compensate for the error from the harmonic oscillation approximation, the peak positions shown here were adjusted using an empirical eq (1.0353*x* – 244 where *x* is the peak position from DFT) found in the previous study.^17^ The static polarizability was computed using linear response theory under an applied uniform electric field. Its derivatives with respect to the mass-weighted normal mode coordinates were evaluated using a three-point numerical differentiation approach.^17,29^ The hyperpolarizability in terms of polarizability and dipole derivatives was calculated under the short-time approximation.^17,29^ The SFG intensities of selected modes were calculated using the algorithm described in our previous publications.^17,29,31^

## Results and Discussion

### Distinguishing Reactant and Product Species of the Reaction between Ph-NCO and Surface OH Groups

Although DFT predictions of absolute vibrational frequencies are subject to systematic errors due to functional and basis set approximations,^32^ the relative ordering of normal modes and their computed hyperpolarizabilities remain useful indicators for interpreting experimentally observed spectral trends.^17,33^ In this study, we used DFT calculations to predict key differences in vibrational spectral features of the phenyl (Ph) ring with isocyanate versus urethane side groups (Figure 1). The former was expected to arise from “physisorbed” reactant molecules remaining at the surface, whereas the latter was attributed to the chemisorbed product formed by the reaction between Ph-NCO and surface OH groups. These theoretical calculations were necessary because no prior study could be found in the literature that describes the effects of molecular orientation and chemical environment on vibrational spectral features of the phenyl group. The transient polarizability and dipole moment tensors predicted from DFT calculations for Ph-NCO and phenyl urethane are shown in Table S1 in the Supporting Information.

**Figure 1:**
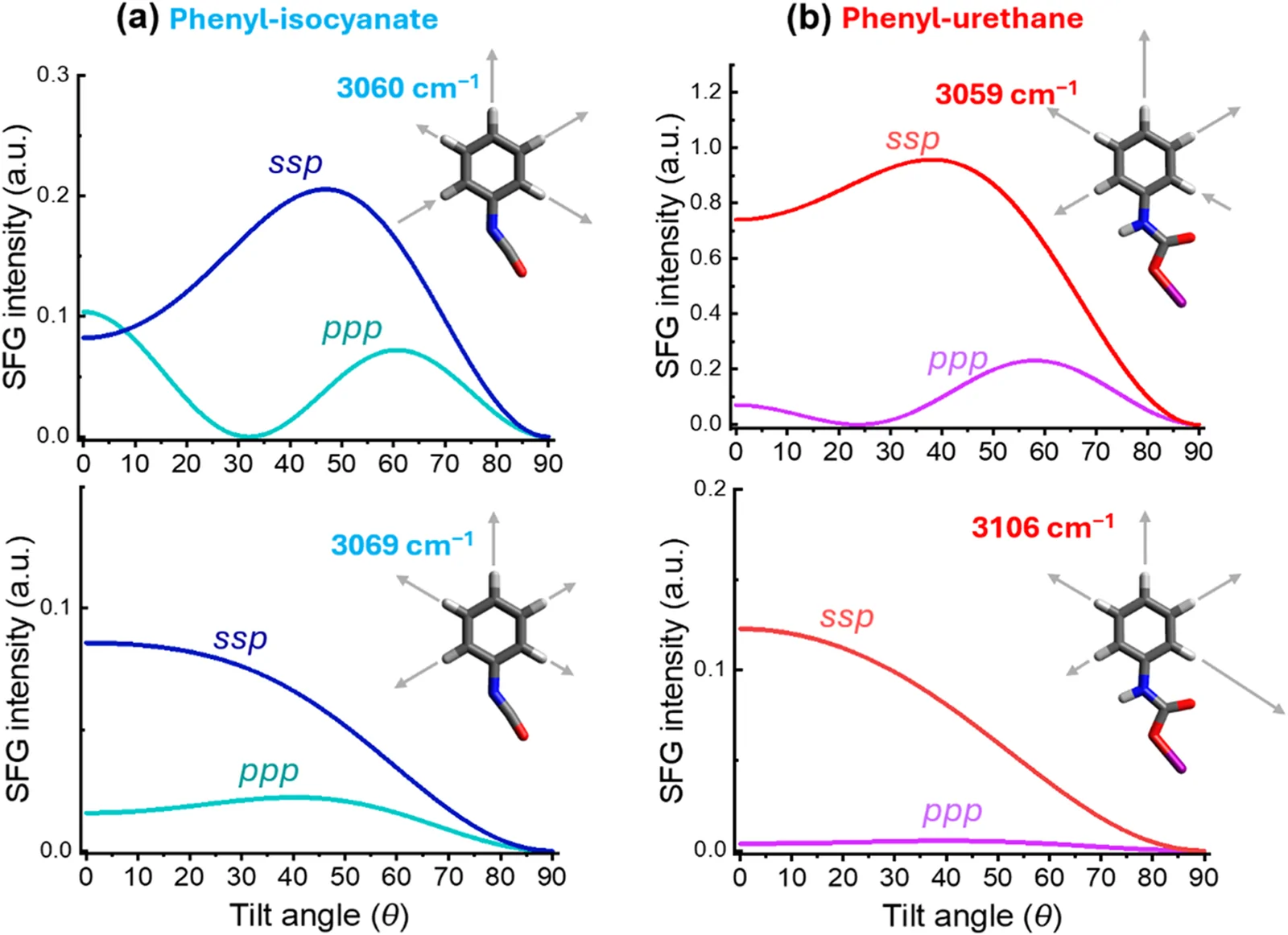
DFT-predicted peak position and *ssp*- and *ppp*-SFG intensities of two main C–H symmetric stretch modes of the phenyl group (resembling the *v*_2_ mode under the *C*_2*v*_ local symmetry) calculated for (a) phenyl isocyanate and (b) phenyl urethane as a function of tilt angle (θ), where θ is defined as the angle between the *C*_2*v*_ axis of the phenyl group (without considering the substituent) and the surface-normal direction. The vibrational motions of C–H bonds are marked with arrows. In the case of urethane, the surface atom to which the single-bond oxygen of the carbamate group is attached is modeled with an iodine atom that does not couple due to a large atomic mass difference from other constituent atoms. In the model structure shown on the left, the color code is as follows: dark gray = C, light gray = H, blue = N, red = O, and purple = I. The relative intensity of the *ppp* and *ssp* signals (*I*_*ppp*_/*I*_*ssp*_) of each mode is shown in Figure S1 in the Supporting Information.

Although the phenyl ring itself can be described with the *C*_2*v*_ point group, its C–H vibrations deviate from the symmetry-allowed normal modes of *C*_2*v*_.^17^ This is due to the coupling with vibrations of substituent groups. In our previous DFT study for vibrations of phenyl groups with various substituent groups, it was found that the mode with the strongest SFG intensity is the normal mode with symmetric C–H stretches that resembles the *v*_2_ mode with the A1 symmetry of the *C*_2*v*_ point group.^17^ Thus, we focus on the *v*_2_-like modes of Ph-NCO and phenyl urethane species, which are shown in Figure 1. Each species shows two modes that could be viewed *close* to the conventional *v*_2_ mode of the phenyl group. Hereafter, the dominant peak of each species will be called *symmetric stretch mode* for simplicity—3060 cm^–1^ for Ph-NCO and 3069 cm^–1^ for phenyl urethane.

The two main findings from DFT calculations are as follows. First, the position of the symmetric stretch peak of phenyl urethane is slightly higher than that of Ph-NCO. This means that the main SFG peak position of the phenyl group will blue-shift upon reaction of the isocyanate group with the surface hydroxyl group. The change in the electronic structure of the substituent group (from −N=C=O to −NH–C­(=O)–O−), as well as the coupling with the N–H stretch, must be the main cause for this slight blueshift.

Second, the relative SFG intensity ratio of this main peak in the *ppp* and *ssp* polarizations (i.e., *I*_*ppp*_/*I*_*ssp*_) increases as the tilt angle (θ) of the phenyl group with respect to the surface-normal direction increases (see Figure S1 in the Supporting Information). The only exception would be the case where the unreacted Ph-NCO aligns the phenyl ring along the surface-normal direction. When θ is larger than 25–30° from the surface-normal direction, the *I*_*ppp*_/*I*_*ssp*_ ratio increases as the phenyl ring tilts more toward the surface. If the symmetric stretch peak of the phenyl group follows the A1 symmetry of the *C*_2*v*_ point group, the *I*_*ppp*_/*I*_*ssp*_ ratio remains unaltered with θ because their effective susceptibilities, χ_*ppp*_^(2)^ and χ_*ssp*_^(2)^, have the same θ-dependence.^17^ In an SFG experiment, it is difficult to compare the measured intensity to the theoretically calculated intensity; but, the relative intensity of the same mode can readily be compared with the trend predicted from theory. Thus, based on DFT calculation results, the change in the *I*_*ppp*_/*I*_*ssp*_ ratio of the symmetric stretch mode of phenyl urethane species can be used as a *qualitative* descriptor for the phenyl group tilt angle.

### Reaction of Surface −OH Group with Ph-NCO

We first tested the feasibility of differentiating the surface reactivity through reactions with Ph-NCO using a silica (fused quartz) surface. The abundance of surface hydroxyl (silanol) groups on the silica surface was controlled by surface cleaning and treatment with HMDS. The difference in surface OH group was confirmed by measuring the water contact angle (θ_water_).^34^ Figure 2 shows the SFG spectra of the clean silica surface with θ_water_ < 10° and the HMDS-treated silica surface with θ_water_ ≈ 75°, which were exposed to Ph-NCO for 10 min at room temperature in two different conditions: (i) nitrogen gas stream saturated with Ph-NCO vapor (partial pressure ≈ 1500 mTorr) and (ii) organic solution consisting of Ph-NCO/NMP = 1:20. Hereafter, the former condition will be called vapor-phase reaction and the latter is solution-phase reaction.

**Figure 2:**
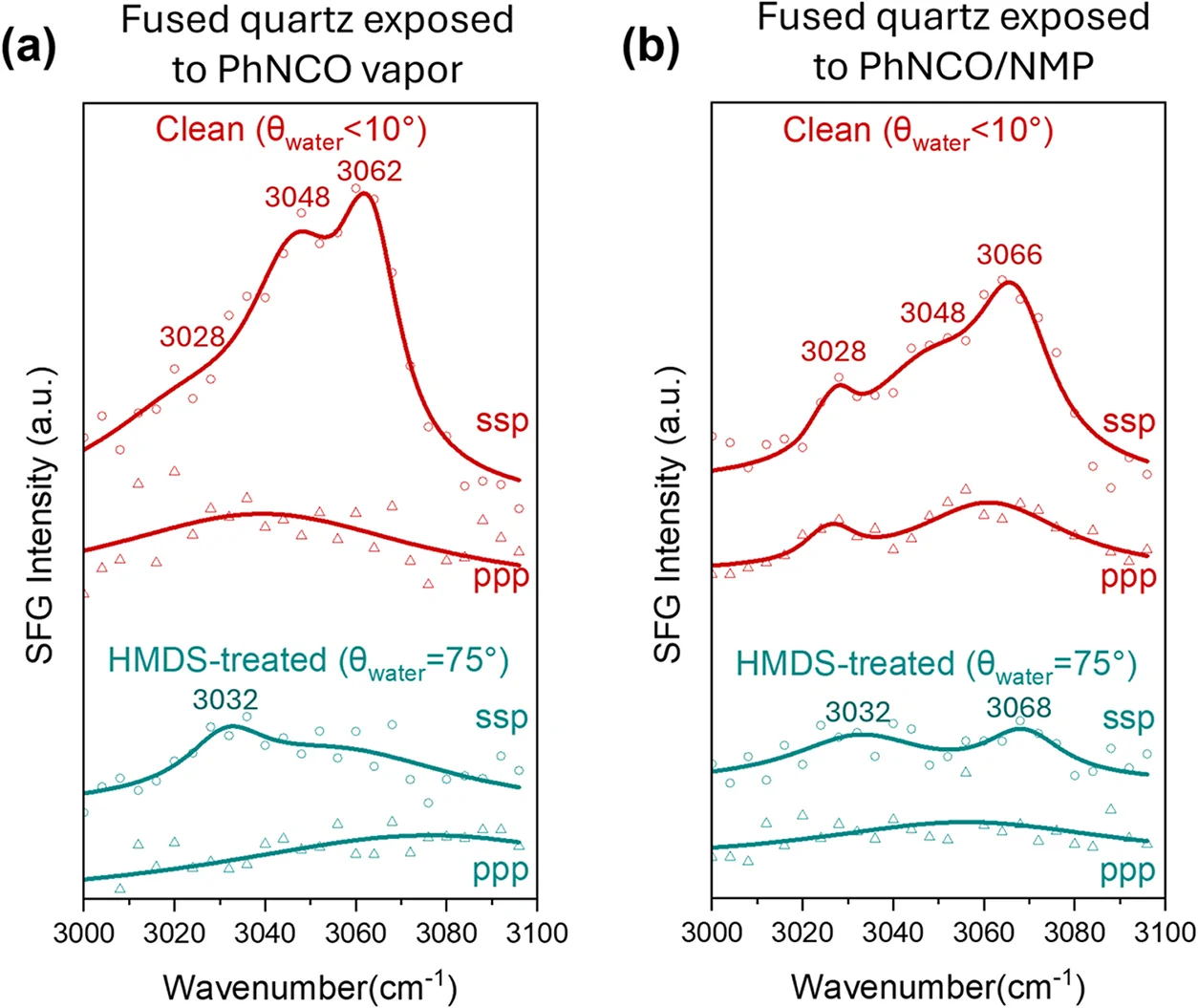
SFG spectra of (a) Ph-NCO vapor and (b) Ph-NCO in NMP adsorbed on clean fused quartz (water contact angle θ_water_ < 10°) and hydrophobized HMDS-treated quartz (θ_water_ ≈ 75°). SFG measurements were done with *ssp* and *ppp* polarization configurations, and the spectra shown here are the average of three measurements with different specimens treated in the same way.

The data shown in Figure 2 suggest that both vapor-phase and solution-phase reactions can differentiate the silica surface chemistries. The hydrophilic surface with a high density of OH groups exhibits much stronger SFG signals of phenyl groups after exposure to Ph-NCO, as compared to the hydrophobic surface in which most −OH groups are converted to −OSi­(CH_3_)_3_ groups through reactions with HMDS. Under both vapor-phase and solution-phase reaction conditions, the *I*_*ppp*_/*I*_*ssp*_ ratio of the main symmetric stretch mode of the product species on the hydrophilic silica surface is quite small. In the vapor-phase reaction case, it is not larger than the noise level. In the solution-phase reaction case, the ratio was around 0.44. On the basis of the DFT calculation results (Figure 1), this small *I*_*ppp*_/*I*_*ssp*_ ratio means that the phenyl group of the reaction product is relatively upright (i.e., away from the silica surface).

The reaction conditions led to some differences in the SFG spectral features of the reaction product species, which is believed to be the phenyl urethane group covalently bound to the silica surface. The main symmetric stretch peak position is slightly different in the SFG spectra of the hydrophilic surfaces reacting with Ph-NCO under two different conditions: 3062 cm^–1^ for the vapor-phase-reacted surface and 3066 cm^–1^ for the solution-phase-reacted surface. At this time, it is not clear what caused this difference. It is possible that, under the vapor-phase reaction conditions, a small amount of residual water molecules strongly adsorbed on the clean silica surface may have reacted with Ph-NCO. If so, the phenyl urea species can be formed, which may have a slightly different peak position for the symmetric phenyl stretch mode than the phenyl urethane species. Also, it is possible that a trace amount of unreacted Ph-NCO species could remain during the SFG analysis since its vapor pressure is extremely low. In contrast, under the solution-phase reaction conditions, any residual water physisorbed on the silica surface would have been removed immediately from the silica surface since water is highly soluble in NMP. Also, any unreacted Ph-NCO residue would have been removed during the rinsing step after the reaction. Therefore, the detected signal is mostly from the phenyl urethane species chemisorbed to the silica surface.

Although water-related side reactions were minimized under the controlled dry environment of this study, residual water strongly bound to the surface can act as a competing reactant toward isocyanates, particularly on highly hydrophilic surfaces such as quartz. In contrast, the clearcoat surfaces investigated here remain substantially less hydrophilic than quartz, suggesting that reactions involving surface OH groups are likely to be the dominant pathway under the conditions studied. Nevertheless, water-induced side reactions cannot be completely excluded. Because the present study was not designed to systematically investigate the competition between water and surface OH groups for reaction with Ph-NCO, the relative contributions of these pathways cannot be determined quantitatively. Any urea-containing species that may form could contribute to variations in the observed peak positions and intensities.

### Reaction of Clearcoat Surface with Ph-NCO Vapor

The characterization methods frequently used to check the surface chemistry change include X-ray photoelectron spectroscopy (XPS) and surface energy measurement. We have conducted XPS analyses of the clearcoat surfaces before and after Ph-NCO reactions, but there were no discernible changes observed (see Figure S2 in the Supporting Information). In contrast, the surface energy measurement showed some contrast. Figure 3 compares the dispersive and polar components of the surface energy of the clearcoat films subjected to aqueous pretreatment at pH 1, 7, and 13, followed by exposure to Ph-NCO vapor. The surface energy of the pristine clearcoat surface is about 38 mJ/m^2^, which is close to that of hydrophobic poly­(methyl methacrylate) (PMMA). Upon pretreatment with acidic or basic aqueous solutions, the dispersive component of surface energy does not change much, but the polar component increases significantly (Figure 3). In contrast, the pH 7 solution does not cause any noticeable change in surface energy. These results indicate that the hydrolysis of the methoxysilyl group to the silanol group does not occur readily upon contact with neutral water and requires acidic or basic catalysis (Scheme 1).^12^

**Figure 3:**
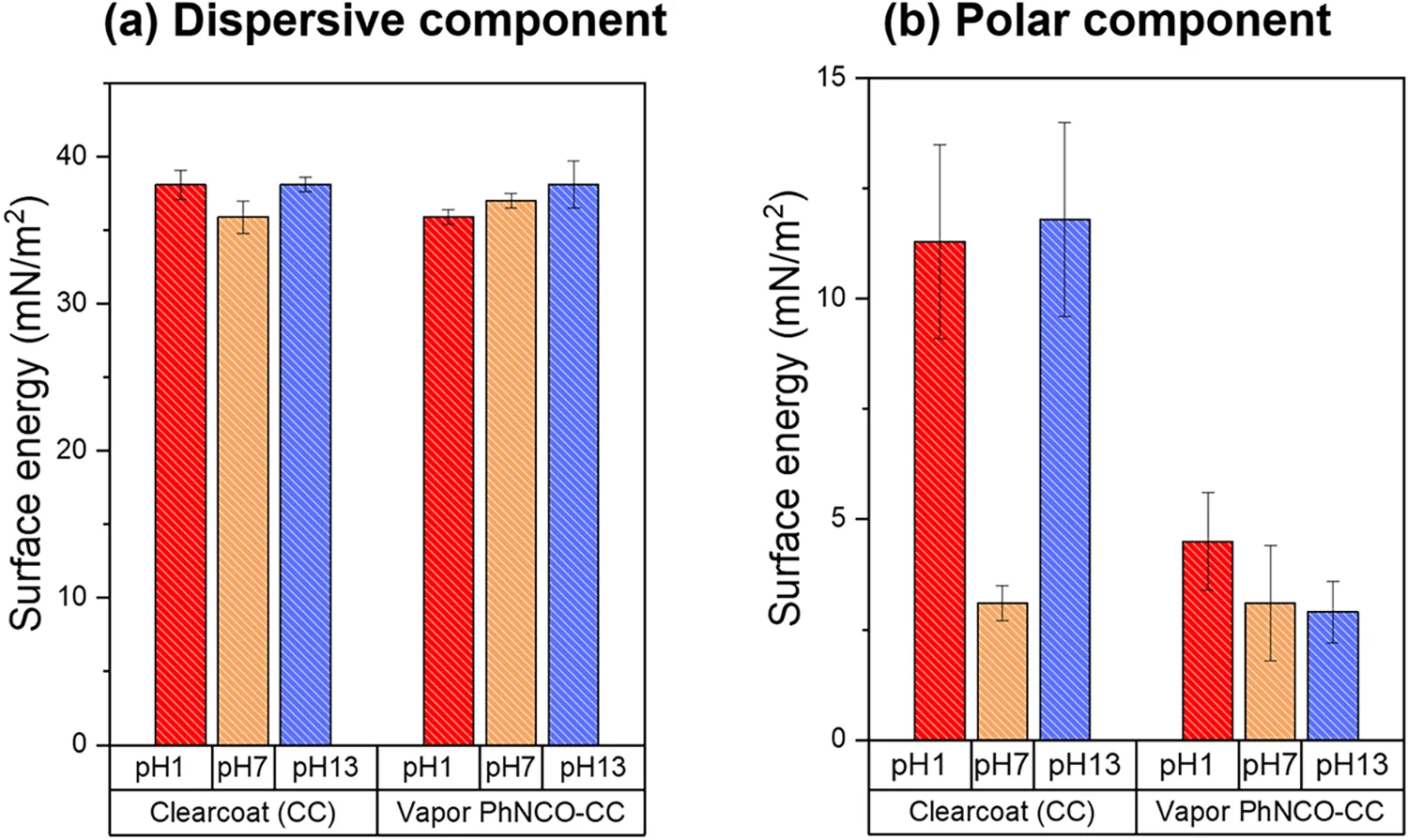
(a) Dispersive and (b) polar components of surface energy for clearcoat (CC) treated with pH 1, 7, and 13 aqueous solutions, followed by exposure to Ph-NCO vapor. The error bar is the standard deviation from 3 measurements.

Upon exposure to the Ph-NCO vapor, the polar components of surface energy of the acid- and base-pretreated surfaces decrease to values comparable to those of the pristine surface and the surface pretreated with pH 7 aqueous solution. These changes imply that the surface-exposed silanol groups are converted to phenyl urethane groups. However, the degree of conversion or conformation of reaction products cannot be discussed from the surface energy change alone.

For molecular characterization, vibrational spectroscopy is widely used. Attenuated total reflectance infrared (ATR-IR) spectroscopy is often considered a surface-region sensitive technique. The ATR-IR spectra of clearcoat specimens that were pretreated with aqueous solutions with different pHs and then reacted with Ph-NCO vapor are shown in Figure S3 in the Supporting Information. There was no discernible difference in the ATR-IR spectra of the specimens tested. Since the ATR-IR probe depth is around 600 nm in the C–H stretch region (∼3000 cm^–1^) and 3–4 μm in the C–O and Si–O stretch region (∼1000 cm^–1^),^19^ the data in Figure S3 indicates that the surface treatment effect is confined to the very top surface region, much shallower than the depth that ATR-IR can probe.

As a more “surface-region” sensitive probe, PiFM analysis was conducted for the specimens pretreated with pH 1 and 13 and then exposed to the Ph-NCO vapor, which showed the largest change in surface energy measurements (Figure 3). The sideband detection mode of PiFM probes about 20 nm from the topmost surface, and the direct-modulation mode probes up to ∼1 μm from the surface.^19,23,24^ In addition, PiFM can check the lateral homogeneity of functional group distribution since it has a high lateral resolution (on the order of probe tip diameter). In Figure S4 in the Supporting Information, an example of a PiFM analysis result is shown for the clearcoat sample pretreated with a pH 1 solution and then reacted with Ph-NCO vapor (raw data of other specimens are not shown). The topography image and the intensity map of the most common signal (1722 cm^–1^) did not show any local irregularity within a 1 μm × 2 μm area that required finer analysis. Six different locations were chosen within the imaged area, and the PiF-IR spectra were collected over the full spectral window. Again, other than typical fluctuations due to local variations in tip-sample interaction dynamics, no significant difference was observed among the six PiF-IR spectra collected at different locations. So, the full spectra were averaged for comparison among the specimens treated in different conditions.

Figure 4 exhibits the PiF-IR spectra of the untreated clearcoat specimen, and the specimens pretreated with aqueous solutions with pH 1 and pH 13, followed by exposure to Ph-NCO in the vapor-phase reaction condition. The pH 13-treated specimen shows spectral features nearly identical to the untreated specimen in both surface-sensitive and bulk-sensitive analysis modes. A slight difference is noted in the relative intensities of the stretch regions of the conformationally more flexible C–O and Si–O bonds (1000–1200 cm^–1^) versus the spectral region of the more rigid triazine ring, carbamate, and ester group stretches (1350–1750 cm^–1^). In the previous study comparing the spectra obtained with photothermal atomic force microscopy coupled with IR (AFM-IR) and ATR-IR, a small variance in the relative intensities of these regions was also observed, which could be attributed to minute structural differences in the subsurface region.^12^

**Figure 4:**
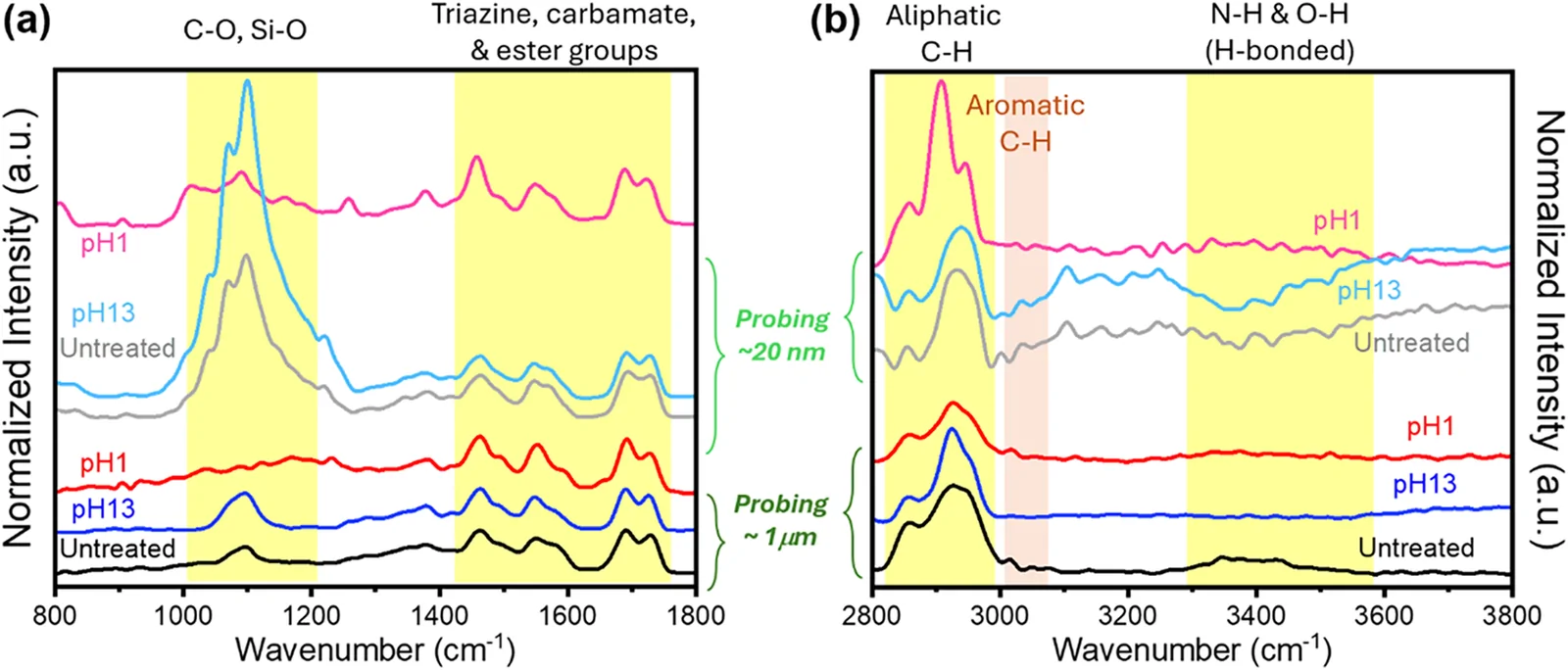
PiF-IR Spectra, collected with surface-sensitive and bulk-sensitive modes, of clearcoat films treated with pH 1 and pH 13 solutions followed by exposure to Ph-NCO vapor, in the wavenumber ranges of (a) 800–1800 cm^–1^ and (b) 2800–3800 cm^–1^. As a reference, the PiF-IR spectra of the untreated pristine specimen are also shown. The surface-sensitive and bulk-sensitive spectra were collected using the sideband mode (probing ∼20 nm from the surface) and the direct-modulation mode (probing on the order of ∼1 μm), respectively. The spectra shown here are the averages of 6 spectra measured at different locations selected from the PiFM image of each sample and normalized with the 1691 and 1722 cm^–1^ peak intensities for comparison. The images and raw spectra of the specimen pretreated with pH 1 followed by Ph-NCO exposure are shown in the Supporting Information (Figure S4).

The specimen treated with pH 1 and then Ph-NCO vapor shows noticeable differences from the untreated and pH 13-pretreated specimens. Moreover, the C–O and Si–O stretch bands, as well as the aliphatic C–H stretch bands, are different in the PiF-IR spectra collected with surface-sensitive and bulk-sensitive modes. These observations indicate that the pH 1-treated specimens exhibit more pronounced changes in the PiF-IR spectra than the pH 13-treated specimens. One possible explanation is the difference between acid- and base-catalyzed hydrolysis and condensation pathways. Under acidic conditions, hydrolysis of methoxysilyl groups may occur more extensively before recondensation takes place, allowing greater rearrangement of the local network structure. Under basic conditions, hydrolysis and condensation are more likely to occur simultaneously, which may limit the degree of network reorganization. However, the present data do not allow the depth or mechanism of these differences to be determined conclusively, and additional depth-sensitive characterization would be required to verify this hypothesis.^35–37^

Although the PiF-IR was able to differentiate chemical changes in the subsurface region that occurred upon pH 1 vs pH 13 pretreatments, it could not detect the phenyl group vibration peaks for the specimens reacted with the Ph-NCO vapor. On the basis of the decrease in surface energy (especially, the polar component) after the vapor-phase reaction with Ph-NCO (Figure 3), it is certain that the surface-exposed Si–OH groups are converted to hydrophobic phenyl urethane groups. This means that PiF-IR could not detect the chemical change of the topmost surface of the clearcoat. This must be because the 20 nm probe depth is still too large to differentiate the chemical species at the topmost surface from those in the subsurface region. To selectively probe the topmost surface chemistry, we used SFG spectroscopy. Because of the noncentrosymmetry requirement of the SFG process, this technique is inherently sensitive to the topmost surface only as long as the subsurface region is amorphous.^19,30,38^

Figure 5 compares the SFG spectra of clearcoat surfaces pretreated with aqueous solutions with three different pHs (1, 7, and 13), followed by the vapor-phase reaction with Ph-NCO. The SFG spectra of specimens treated under other pH conditions are shown in Figure S5a in the Supporting Information. Under neutral conditions (pH 7), the hydrolysis of methoxysilyl groups to silanol groups does not occur readily in at least 10 min of treatment at room temperature (Figure 5a). This is due to the lack of catalytic activity. When the clearcoat surface is pretreated with strong acid (pH 1) or strong base (pH 13) for 10 min, the hydrolysis of methoxysilyl groups occurs readily, forming silanol groups (Scheme 1). Thus, the free OH stretch signal is seen at 3690 cm^–1^ in the *ppp*-SFG spectra of these specimen surfaces (Figure 5a). The free–OH signal is negligible in the *ssp*-SFG spectra. From the SFG theory, we know that the *ssp* polarization selectively detects the surface normal component of vibrational modes, while the *ppp* polarization detects both surface normal and parallel components.^19^ Thus, the absence of the free–OH signal in the *ssp*-SFG spectra means that the free–OH groups exposed at the clearcoat surface after pretreatment with pH 1 and 13 solutions lie predominantly parallel to the surface. This is quite different from the free–OH group present at the silica surface, which shows strong peaks in both *ssp*- and *ppp*-SFG spectra.^39–41^ This difference must be due to the flexibility of polymer chains exposed at the clearcoat surface, allowing the OH group to tilt toward the surface. Although the free–OH groups appear to adopt a predominantly surface-parallel orientation on average, the local relaxations of polymer chains exposed at the clearcoat surface allow local segmental motions that can transiently expose OH groups in geometries favorable for reaction with isocyanates. In contrast, at the silica surface, Si–OH groups face along the surface-normal direction because all bridging oxygen (Si–O–Si) bonds are pointing inward to the glass network.

**Figure 5:**
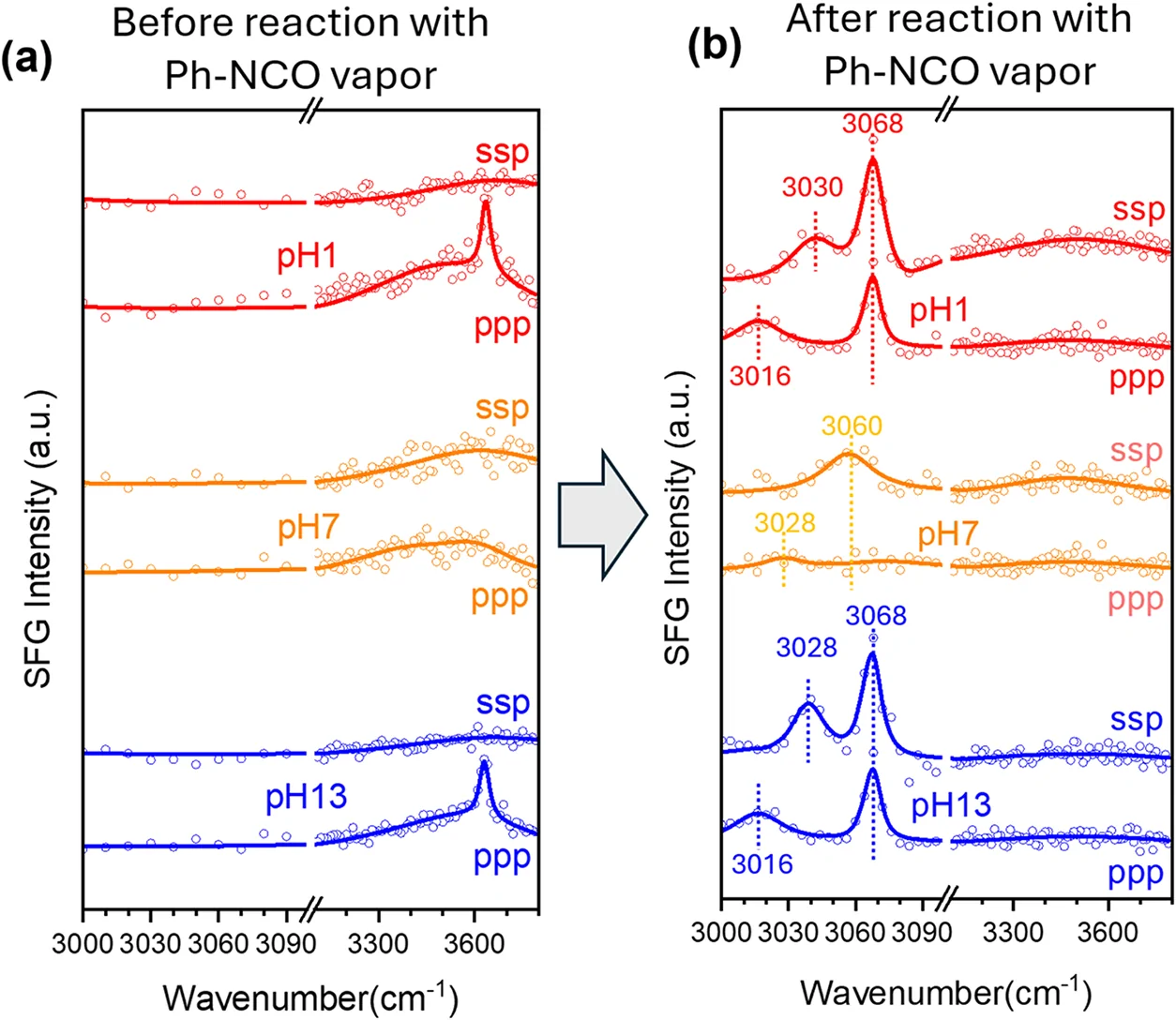
SFG spectra, collected with *ssp* and *ppp* polarizations, of clearcoat films treated with pH 1, 7, and 13 solutions: (a) before and (b) after exposure to Ph-NCO vapor for 10 min at room temperature. The spectra shown here are the averages of three spectra measured with three different samples. The SFG spectra of specimens pretreated with pH 3, 4, 10, and 11, followed by exposure to Ph-NCO vapor, are shown in Figure S5a in the Supporting Information.

Upon exposure of the pH 7-pretreated clearcoat surface to the Ph-NCO vapor, the growth of the phenyl signal in the SFG spectra taken after purging with dry nitrogen is relatively insignificant (Figure 5b). Similarly, the growth of the phenyl signal on the pH 3, 4, 10, and 11-pretreated clearcoat surfaces after the Ph-NCO vapor is only marginal (Figure S5a). The peak positions of the weak symmetric stretch bands observed for these specimens are ∼3060 cm^–1^, which could be attributed to a small amount of Ph-NCO species physisorbed on the clearcoat surface (or phenyl urea formed by their reaction with ambient water molecules during the sample transfer and SFG measurement). In contrast, the pH 1- and 13-pretreated clearcoat surfaces show a significant growth of the phenyl signal at 3068 cm^–1^ while the free–OH signal disappears in the *ppp*-SFG spectra (Figure 5b). Its peak position is even higher, and its peak width is narrower than the ones seen on the hydrophilic silica surface after exposure to Ph-NCO vapor (Figure 2). These phenyl groups detected with SFG on the clearcoat surface indicate that the surface-parallel OH groups on the pH 1- and 13-pretreated clearcoat surface readily react with Ph-NCO impinging from the gas phase.

The *I*_*ppp*_/*I*_*ssp*_ ratio of the 3068 cm^–1^ peak is around 0.9; this large ratio is most consistent with a predominantly surface-parallel orientation of the phenyl ring among the multiple mathematical solutions allowed by the nonmonotonic curve (see Figure S1b). This is quite different from the very small *I*_*ppp*_/*I*_*ssp*_ ratio of the 3062 and 3066 cm^–1^ peaks observed on the hydrophilic silica surface after reaction with Ph-NCO (Figure 2). Again, this must be due to the difference in the free–OH group orientation between the clearcoat surface (predominantly surface-parallel) and the silica surface (closer to surface normal).

### Comparison of Vapor-Phase vs Solution-Phase Reactions of Clearcoat Surface

The commercial urethane adhesive evaluated in this study contains solvents that include NMP. So, to understand the effect of NMP on the reaction of the clearcoat surface with Ph-NCO, SFG analysis was employed to find any differences in the phenyl group conformation in the phenyl urethane species on the clearcoat surfaces formed in the vapor-phase vs solution-phase reaction conditions. Figure 6 compares the *ssp*- and *ppp*-SFG spectra of the clearcoat surfaces exposed to Ph-NCO vapor versus Ph-NCO/NMP solution. The full series of the clearcoat surfaces pretreated with various pH conditions is shown in Figure S5b in the Supporting Information.

**Figure 6:**
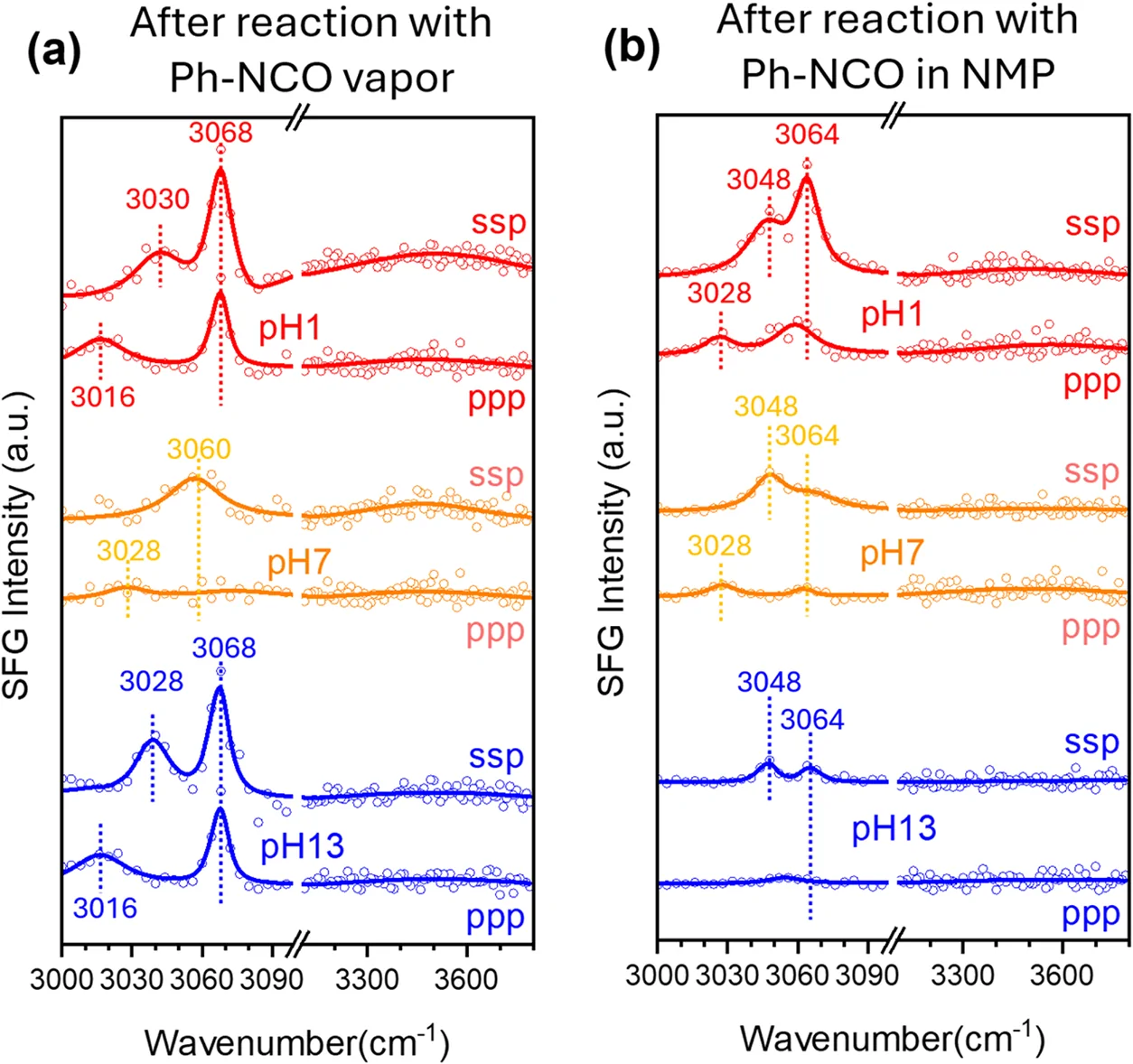
SFG spectra, collected with *ssp* and *ppp* polarizations, of clearcoat films treated with pH 1, 7, and 13 solutions after (a) vapor-phase reaction and (b) solution-phase reaction with Ph-NCO for 10 min at room temperature. The spectra shown here are the averages of three spectra measured with three different samples. The SFG spectra of specimens pretreated with pH 3, 4, 10, and 11, followed by exposure to Ph-NCO vapor, are shown in Figure S5 in the Supporting Information.

The data shown in Figure 6 reveals that the phenyl urethane conformation exposed at the clearcoat surface varies depending on the reaction condition. In the solution-phase reaction, the symmetric stretch peak of the product phenyl group is located at 3064 cm^–1^ (Figure 6b), about 4 cm^–1^ lower than the species formed in the vapor-phase reaction condition (Figure 6a). In the *ssp*-SFG spectrum of the pH 7-pretreated surface, the strongest peak in the phenyl stretch region is seen at ∼3048 cm^–1^, while the symmetric stretch mode at 3064 cm^–1^ is observed with a slightly weaker intensity. Unlike the vapor-phase reaction case which has a strong signal at 3068 cm^–1^, the pH 13-pretreated clearcoat surface exhibits a relatively weak signal at 3064 cm^–1^. Since the solution-phase reacted surfaces were thoroughly rinsed with NMP before drying, it is very unlikely that these phenyl signals are due to residual Ph-NCO reactant at the specimen surface. Ruling out this possibility, the plausible explanation is the conformational difference of phenyl urethane product species exposed at the clearcoat surface.

Although the clearcoat polymer network is highly cross-linked by melamine components (Scheme 1), the clearcoat surface can still swell while in contact with NMP.^12^ Then, the polymer chains between the cross-link points can readily reorient while in contact with NMP and collapse to the conformation with the lowest surface energy upon drying. This hypothesis has been tested and confirmed by analyzing the pH 1- and 13-pretreated specimens before and after contact with the NMP solvent. The *ppp*-SFG spectra collected after NMP exposure show that the free–OH signal disappears, which is accompanied by small changes in the alkyl C–H stretch region in the *ssp*-SFG spectra (Figure 7). The same NMP-induced swelling process is expected for the pH 7-pretreated specimen surface; however, the SFG spectra measured after the NMP treatment are almost identical to those measured before the NMP treatment. This is because the surface energy of the pH 7-pretreated specimen is already at the lowest possible state (Figures 3 and S6).

**Figure 7:**
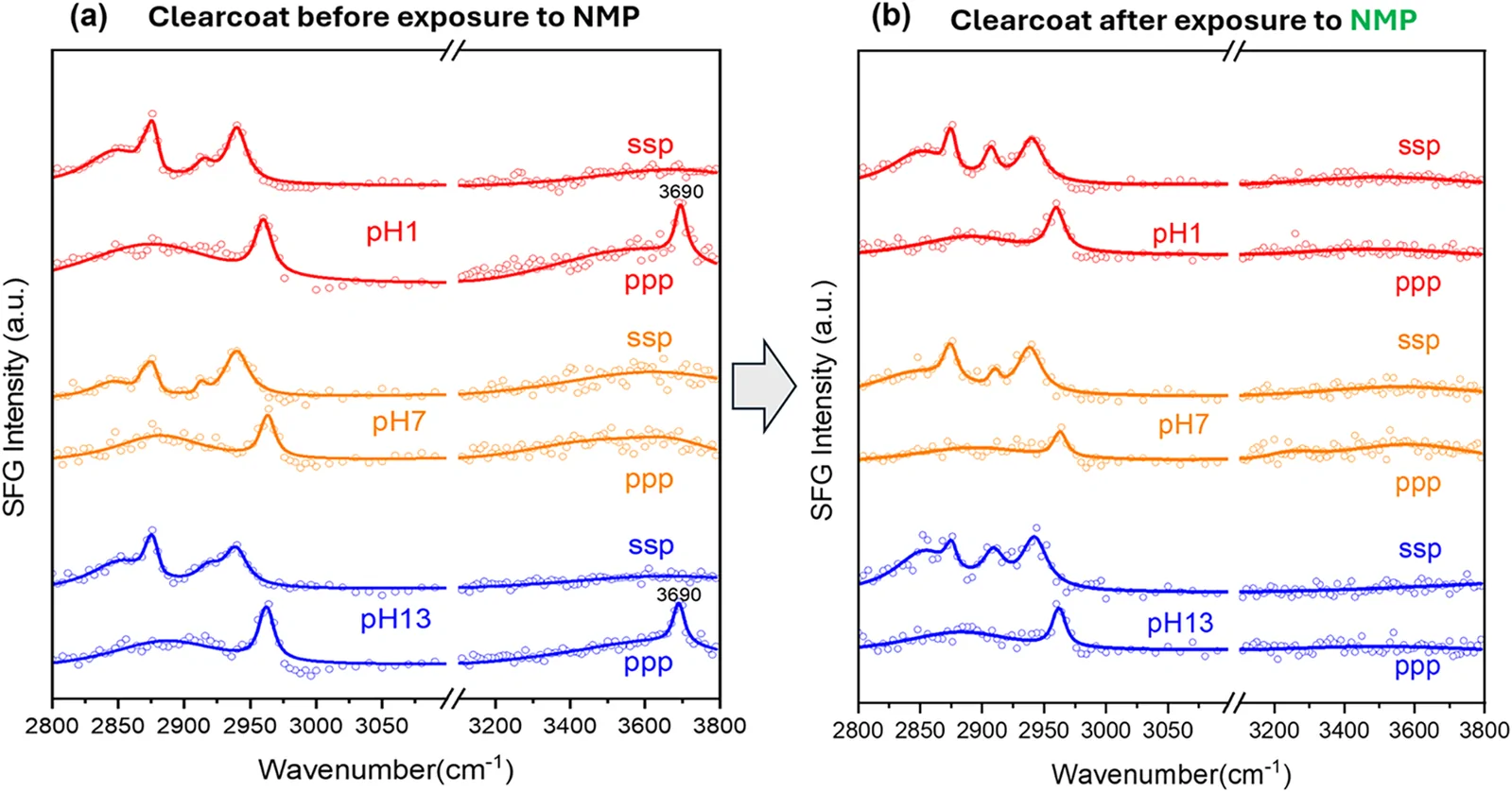
SFG spectra, collected with ssp and ppp polarizations, of clearcoat films treated with pH 1, 7, and 13 solutions (a) before and (b) after exposure to NMP for 10 min at room temperature. The spectra shown here are the averages of three spectra measured with three different samples.

The present results are consistent with NMP-induced swelling increasing the accessibility of OH groups beneath the outermost surface; however, the depth of swelling is not directly measured in this study. Likewise, the relationship between the swelling depth and the probe depths of SFG, PiFM, and ATR-IR is not quantified. Therefore, the role of swelling is inferred from the combined spectroscopic observations rather than direct measurement. Specifically, the appearance of phenyl urethane features following solution-phase reaction, together with the spectral changes observed after NMP exposure, suggests increased accessibility of OH groups that are not readily accessible under vapor-phase conditions. However, the spatial distribution of these OH groups and the extent of swelling remain unresolved.

Figure 8 plots the *I*_*ppp*_/*I*_*ssp*_ ratio of the symmetric stretch peak of the phenyl group of the phenyl urethane species formed at the clearcoat surface after the reaction with Ph-NCO in vapor-phase and solution-phase conditions. Based on the peak position, the phenyl species detected after the vapor-phase reaction of the pH 3, 4, 7, 10, and 11-pretreated surfaces are likely due to a residual Ph-NCO species physisorbed on the surface. Based on DFT calculation results (Figure 1), the change in the *I*_*ppp*_/*I*_*ssp*_ ratio indicates that orientational differences of the physisorbed molecules are present at the clearcoat surface. The large *I*_*ppp*_/*I*_*ssp*_ ratio (∼0.9) for the pH 1- and pH 13-pretreated clearcoat surfaces indicates that the phenyl group of the phenyl urethane product species is predominantly lying parallel to the surface.

**Figure 8:**
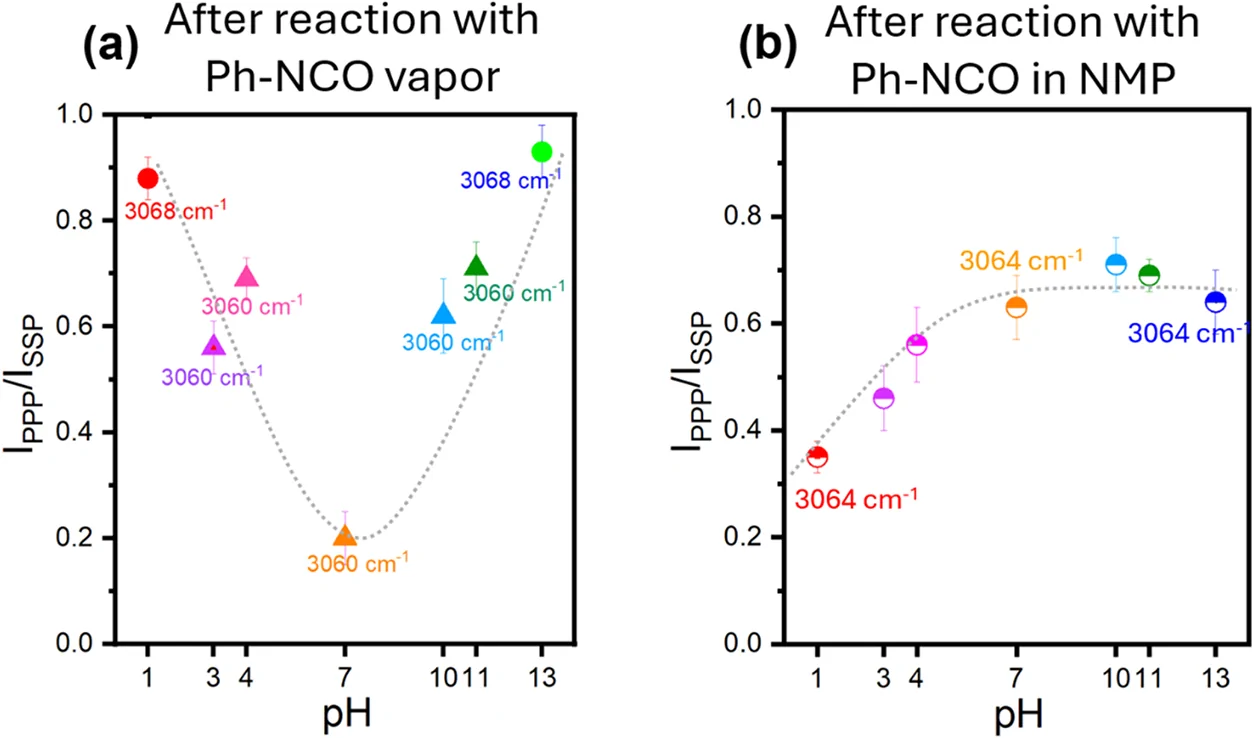
Intensity ratios of the *ppp* vs *ssp* polarization SFG signals (*I*_*ppp*_/*I*_*ssp*_) of the phenyl group of the reaction product formed on the clearcoat films treated with different pH solutions and then undergone (a) vapor-phase reaction with Ph-NCO and (b) solution-phase reaction with Ph-NCO in NMP. The peak positions of the bands analyzed are marked. Dashed lines are included solely to guide the eye and are not intended to imply a continuous relationship between the discrete pH treatment conditions. Error bars represent the standard deviation from three independent measurements.

The orientation analysis based on *I*_*ppp*_/*I*_*ssp*_ assumes an azimuthally isotropic surface population and a dominant average orientation of the phenyl group. Under these assumptions, comparison with the DFT-predicted tilt-angle dependence of *I*_*ppp*_/*I*_*ssp*_ provides qualitative information about the relative phenyl orientation. However, because the calculated curves are not monotonic, a given experimental ratio may correspond to more than one tilt angle. Therefore, orientation values should be regarded as approximate ensemble-averaged trends rather than as unique molecular tilt angles. In addition, the clearcoat surface is chemically heterogeneous, and multiple local environments for phenyl urethane formation may broaden the orientational distribution and influence the apparent *I*_*ppp*_/*I*_*ssp*_ ratios.

In the case of the solvent-phase reaction, the phenyl group conformation of the phenyl urethane species appears to vary depending on the pretreatment condition. While the pH 7-pretreated surface does not readily react with Ph-NCO vapor (as implied by the 3060 cm^–1^ peak position of the phenyl symmetric stretch band), it reacts readily with Ph-NCO in the presence of NMP solvent (a 4 cm^–1^ blueshift as compared to the vapor-phase reaction case). This can be attributed to the NMP-induced swelling of the clearcoat surface, which makes the subsurface OH groups available for reaction with Ph-NCO. For the specimens pretreated with neutral and basic solutions (pH 7, 10, 11, and 13), the tilt angle of the phenyl group is found to be smaller than that of the vapor-phase reaction product (since *I*_*ppp*_/*I*_*ssp*_ is 0.6–0.7 in Figure 6b). In the case of specimens pretreated with acidic solutions, the *I*_*ppp*_/*I*_*ssp*_ ratio decreases as the pretreatment acidity increases (pH 4 → 3 → 1). This means a decrease in the phenyl group tilt angle. This difference could be attributed to a different subsurface structure as inferred from the PiF-IR analysis (Figure 4).

Overall, this study provides molecular-level insight into the mechanisms governing urethane adhesive bonding to automotive clearcoat surfaces. The results of this model study show that effective interfacial bonding depends strongly on the presence and accessibility of reactive OH groups at or near the clearcoat surface. Surface pretreatments with acidic or basic solutions influence both the availability and orientation of these OH groups, thereby controlling their reactivity toward Ph-NCO. In addition, NMP, a common solvent present in urethane adhesives, promotes swelling of the clearcoat surface and exposes reactive sites beneath the outermost layer, enabling reactions that are not accessible in the absence of organic solvents. The molecular orientation of functional groups attached to the newly formed urethane linkage varies depending on the surface-exposed OH groups and the swelling of the clearcoat surface during the reaction. Overall, this study provides molecular-level insight into the reactivity of clearcoat OH groups toward isocyanates. These findings clarify how surface chemistry and solvent-induced restructuring govern interfacial urethane-forming reactions in this model system.

## Conclusions

This study elucidates the key factors that control the reactivity of automotive clearcoat surfaces toward a model isocyanate species. The results support the hypothesis that urethane bond formation at the clearcoat–adhesive interface depends strongly on the presence of free OH groups that are accessible to Ph-NCO. Clearcoat surfaces with limited accessibility of reactive OH groups exhibit low reactivity toward isocyanates, which is expected to correlate with poor interfacial bonding. In addition, NMP, a solvent commonly present in commercial urethane adhesives, promotes interfacial reactivity by inducing swelling of the clearcoat surface and increasing the accessibility of hydroxyl groups within the subsurface region. Together, these findings demonstrate that interfacial urethane formation in this model system is governed by the combined effects of surface chemical functionality and solvent-induced surface restructuring. Although Ph-NCO serves as a useful model isocyanate for probing interfacial reactivity, the present study does not evaluate commercial adhesive formulations. Therefore, the conclusions should be interpreted as mechanistic insight into urethane-forming reactions at the clearcoat interface rather than a direct assessment of adhesive bonding performance.

## Supplementary Material



## Notes

Transient polarizability and dipole moment tensors obtained from DFT calculations (Table S1); predicted *I*_*ppp*_/*I*_*ssp*_ ratios for symmetric stretch modes of phenyl isocyanate and phenyl urethane (Figure S1); XPS spectra of clearcoat samples under different pH pretreatments after exposure to phenyl isocyanate (Figure S2); ATR-IR spectra of treated and untreated clearcoat films (Figure S3); PiFM and PiF-IR analysis of clearcoat surfaces including topography and spatially resolved spectra (Figure S4); SFG spectra of clearcoat films under various pH conditions for vapor- and solution-phase reactions (Figure S5); dispersive and polar components of surface energy for treated clearcoat films (Figure S6); and detailed computational and experimental methodologies and supporting references (PDF)
