# Supplementary material for: Understanding Surface Reaction of Acrylosilane–Melamine Clearcoat with Phenyl Isocyanate
Source: Langmuir. 2026 Aug 6;42(32):23386–97. doi: 10.1021/acs.langmuir.6c02112 (PMC13492361; doi:10.1021/acs.langmuir.6c02112)
Supplement: Supplementary file 1 [file la6c02112_si_001.pdf]

Supporting information

Understanding Surface Reaction of Acrylosilane-Melamine Clearcoat with Phenyl Isocyanate

Yiwen Guo,[1] Jihyeong Ryu,[1] Juseok Choi,[1] Sibing Chen,[2] Xing Chen,[2] Carl Brent Douglas,[3] Delson Trindade,[3] Yongqing Huang,[3] Yun Jing,[4] Padraic O'Reilly,[4] Sung Park,[4] and Seong H. Kim[1]*

1. Department of Chemical Engineering and Materials Research Institute, Pennsylvania State University, University Park, PA 16802, USA
2. Institute of Molecular Plus, School of Chemical Engineering and Technology, Tianjin University, Tianjin 300192, P. R. China
3. Global Innovation Center, Axalta Coating Systems, Philadelphia, PA 19112, USA
4. Molecular Vista, 6840 Via del Oro, Suite 110, San Jose, CA 95119, USA

* Corresponding author: shk10@psu.edu

Number of pages: 7

Number of figures: 6

Number of schemes:0

Number of tables: 1

Table of Contents

- **Table S1:** Transient polarizability and dipole moment tensors obtained from DFT.
- **Figure S1:** I_{ppp}/I_{ssp} ratio of two symmetric stretch modes predicted from DFT calculations for (a) Ph-isocyanate and (b) Ph-urethane.
- **Figure S2:** XPS spectra in the C 1s, N 1s, and O 1s regions for untreated clearcoat and clearcoat pretreated at various pH levels and subsequently exposed to phenyl isocyanate (Ph-NCO) vapor.
- **Figure S3:** ATR-IR spectrum of untreated clearcoat and clearcoat pretreated at various pH levels and subsequently exposed to phenyl isocyanate (Ph-NCO) vapor.
- **Figure S4:** PiFM topography, chemical map ($1722\ \mathrm{cm}^{-1}$), and surface/bulk-sensitive PiF-IR spectra of pH 1-treated clearcoat after exposure to phenyl isocyanate vapor.
- **Figure S5:** SFG spectra (ssp and ppp polarizations) of clearcoat films pretreated at pH 1–13 after (a) vapor-phase and (b) solution-phase reactions with phenyl isocyanate.
- **Figure S6:** Dispersive and polar components of the surface energy of clearcoat films pretreated at pH 1, 7, and 13 following vapor-phase and solution-phase reactions with phenyl isocyanate.

Table S1. Transient polarizability and dipole moment tensors obtained from DFT calculations. The (3×3) matrix represents the polarizability tensor, α_{ij}, and the (3×1) matrix shows the dipole tensor, μ_k, of each mode. The tensor product of these two gives the hyperpolarizability, $\beta_{ijk} = \alpha_{ij} \otimes \mu_k$ (where i, j, k are the principal axes of the molecular coordinate), that can be used for calculation of the second-order susceptibility, $\chi_{IJK}^{(2)}$ (where I, J, K are the principal axes of the laboratory coordinate), contributing to the SFG signal in *ssp* and *ppp* polarization combinations. The procedural details are fully described in Refs. 1, 2, and 3.

Molecule	wavenumber	$\alpha_{ij} \otimes \mu_k$
Ph-isocyanate	**3060 cm^{-1}**	$\begin{pmatrix} 3.52 & 0 & -0.82 \\ 0 & -0.03 & 0 \\ -0.82 & 0 & 9.82 \end{pmatrix} \otimes \begin{pmatrix} -0.18 \\ 0 \\ -0.28 \end{pmatrix}$
	3069 cm^{-1}	$\begin{pmatrix} 12.69 & 0 & 1.31 \\ 0 & -0.10 & 0 \\ 1.31 & 0 & 8.24 \end{pmatrix} \otimes \begin{pmatrix} 0.15 \\ 0 \\ -0.08 \end{pmatrix}$
Ph-urethane	**3069 cm^{-1}**	$\begin{pmatrix} 8.39 & 0 & 0.32 \\ 0 & 0.08 & -0.02 \\ 0.32 & -0.02 & 13.94 \end{pmatrix} \otimes \begin{pmatrix} -0.07 \\ 0 \\ -0.35 \end{pmatrix}$
	3113 cm^{-1}	$\begin{pmatrix} 6.26 & 0 & 2.05 \\ 0 & -0.15 & -0.01 \\ -0.82 & -0.01 & 4.88 \end{pmatrix} \otimes \begin{pmatrix} -0.07 \\ 0 \\ -0.12 \end{pmatrix}$

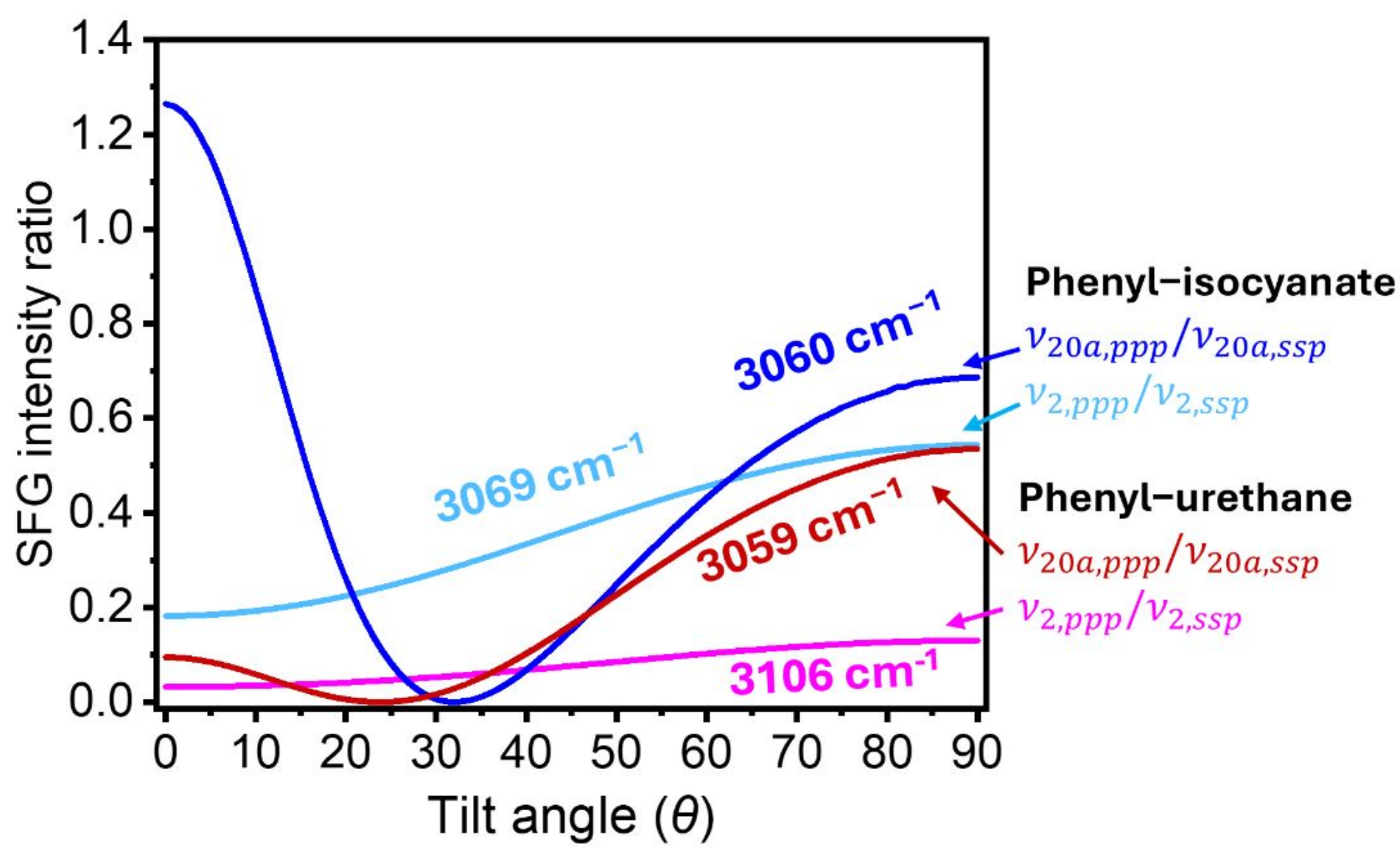

Figure S1. I_{ppp}/I_{ssp} ratio of two symmetric stretch modes predicted from DFT calculations for (a) Ph-isocyanate and (b) Ph-urethane. The dominant peak with the larger intensity is 3060 cm^{-1} in (a) and 3069 cm^{-1} in (b).

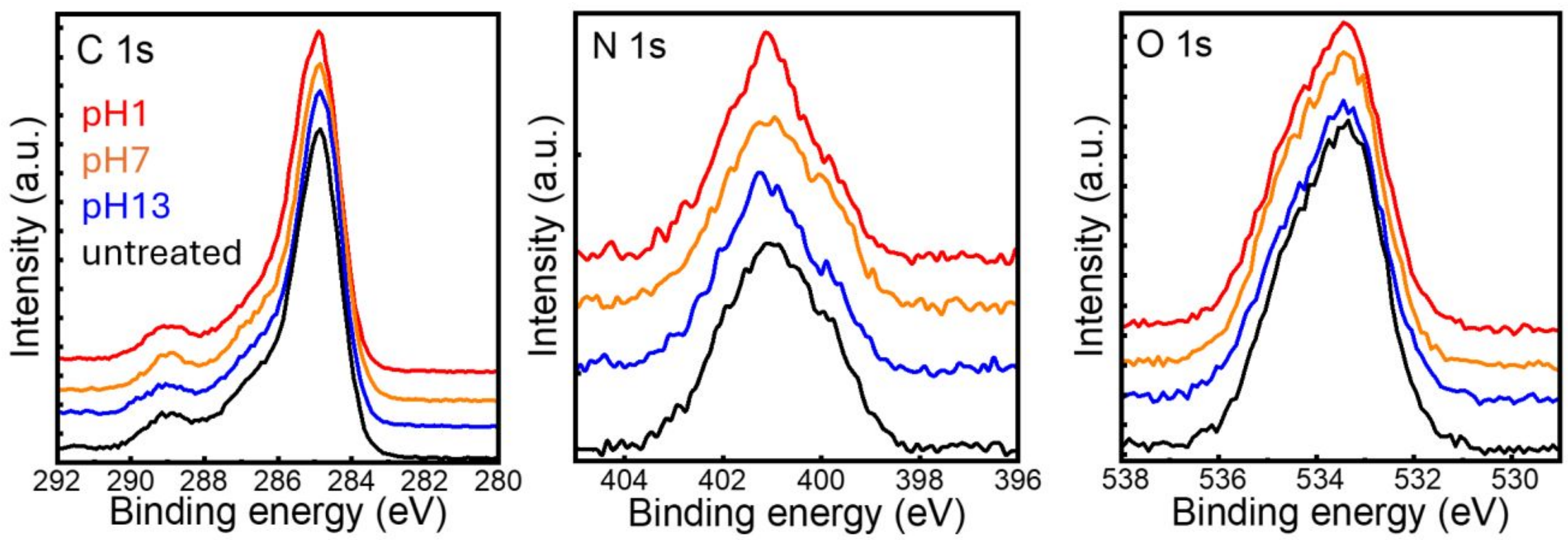

Figure S2. XPS spectra in the C 1s, N 1s, and O 1s regions for untreated clearcoat and clearcoat pretreated at various pH levels and subsequently exposed to phenyl isocyanate (Ph-NCO) vapor.

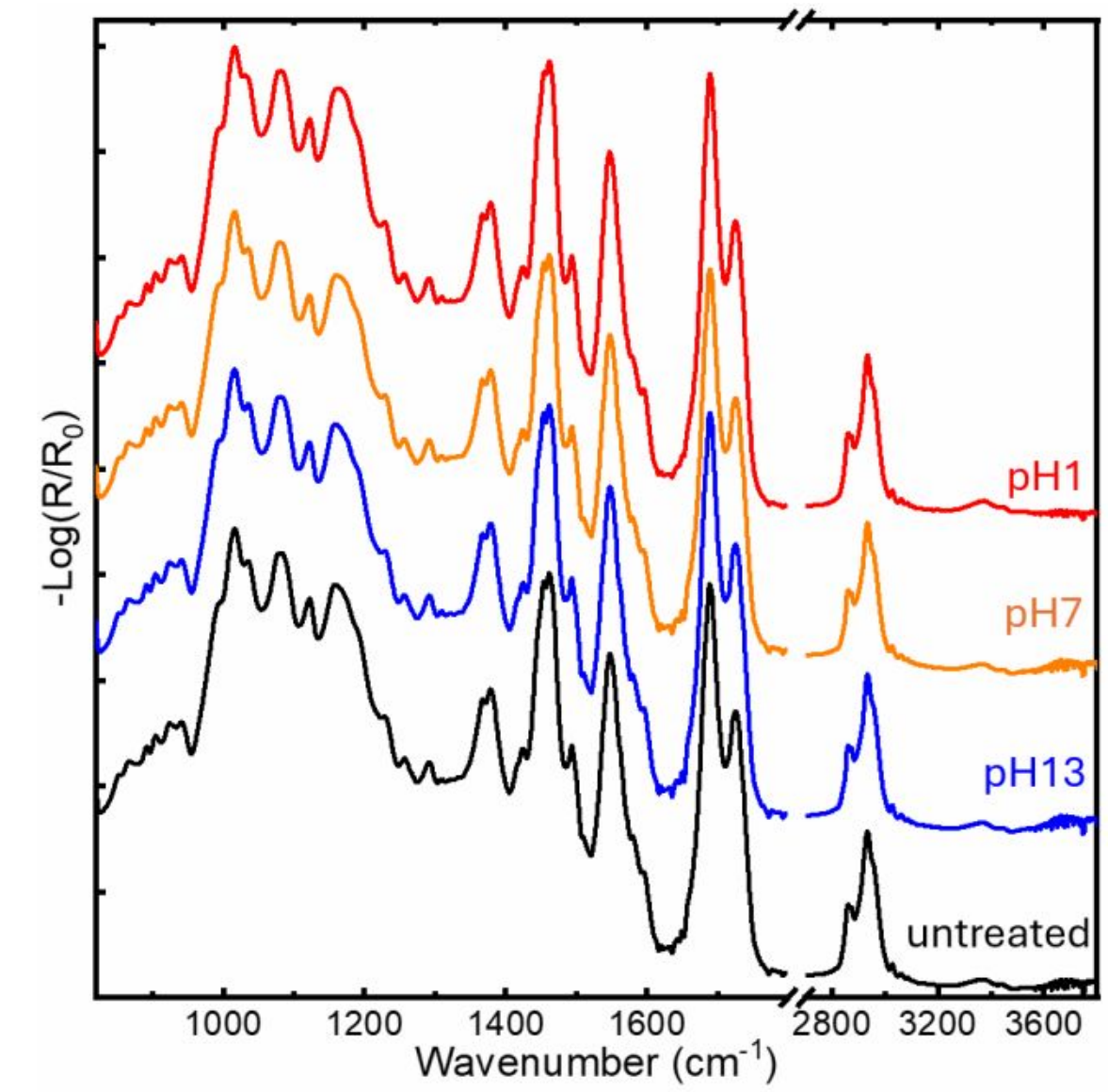

Figure S3. ATR-IR spectrum of untreated clearcoat and clearcoat pretreated at various pH levels and subsequently exposed to phenyl isocyanate (Ph-NCO) vapor.

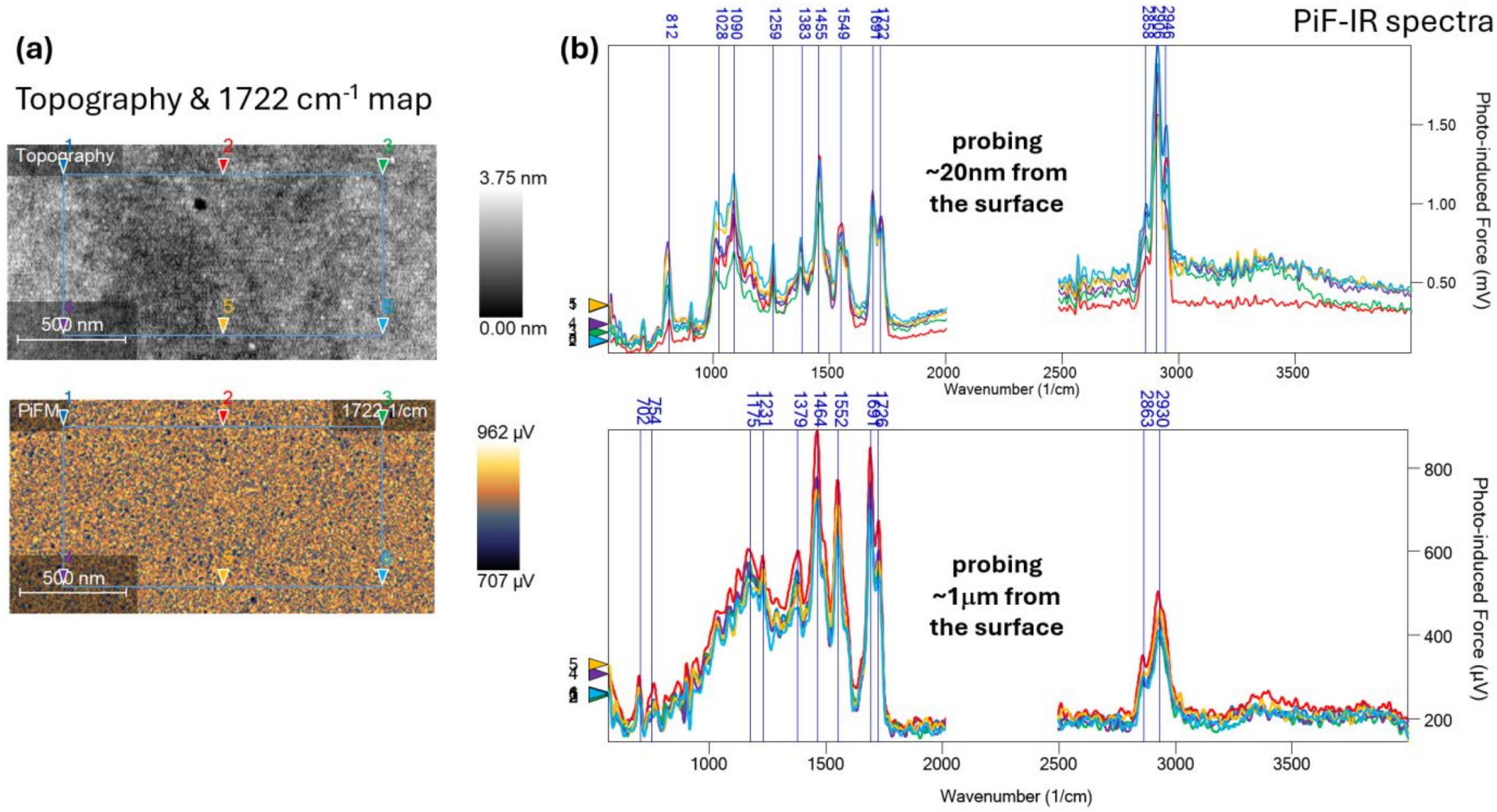

Figure S4. Clearcoat treated at pH 1 and subsequently exposed to phenyl isocyanate (Ph–NCO) vapor, analyzed by photo-induced force microscopy (PiFM) and photo-induced force infrared (PiF-IR) spectroscopy. (a) Representative topography image and corresponding PiFM chemical map acquired at 1722 cm⁻¹ over a 1 × 2 µm² area. Six locations are marked and were selected for the spectral measurements shown in (b) PiF-IR spectra collected at the six indicated positions in (a) using two detection modes: a surface-sensitive mode (probing depth ~20 nm) and a bulk-sensitive mode (probing depth ~1 µm).

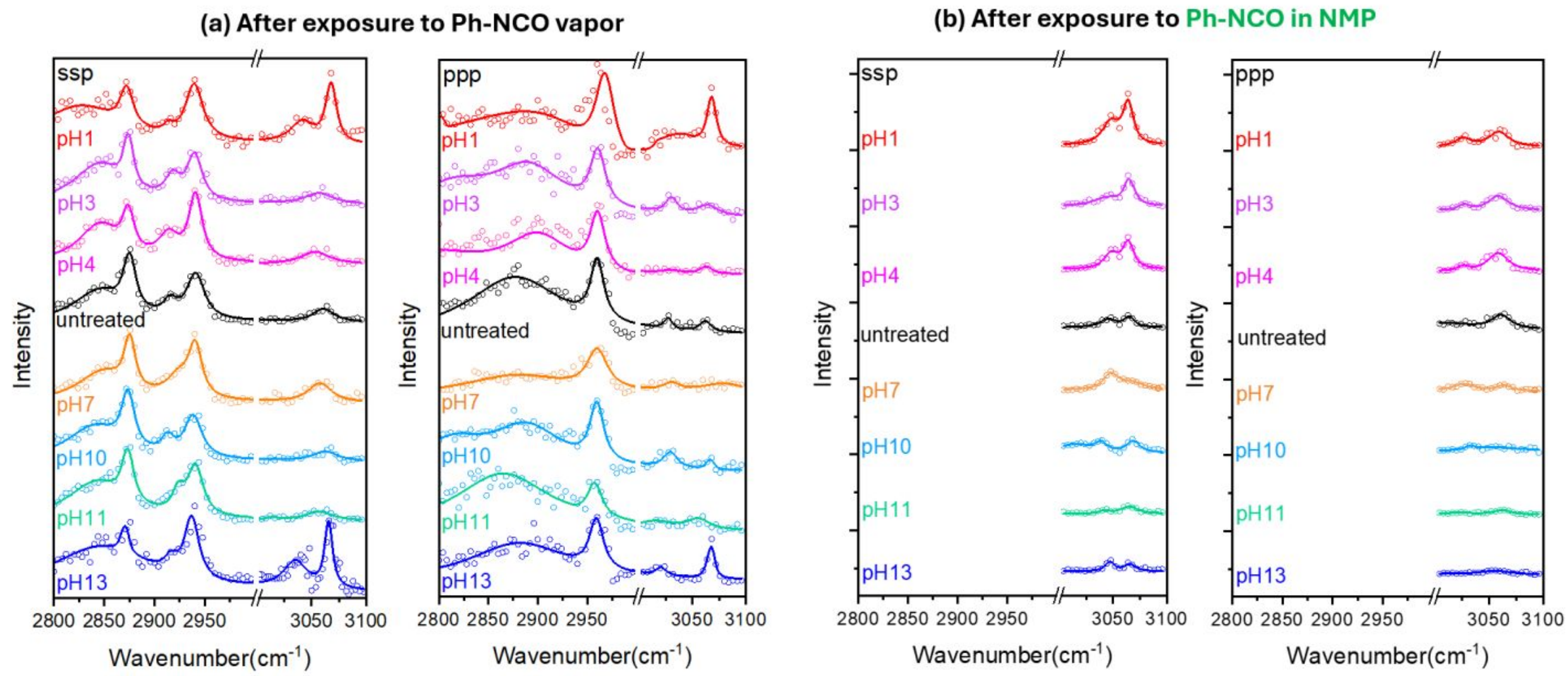

Figure S5. SFG spectra of clearcoat films treated with solutions of pH 1, 3, 4, 7, 10, 11, and 13, collected using ssp and ppp polarization combinations after reaction with phenyl isocyanate (Ph–NCO) for 10 min at room temperature: (a) vapor-phase reaction and (b) solution-phase reaction. The spectra shown represent the average of three measurements obtained from three independent samples.

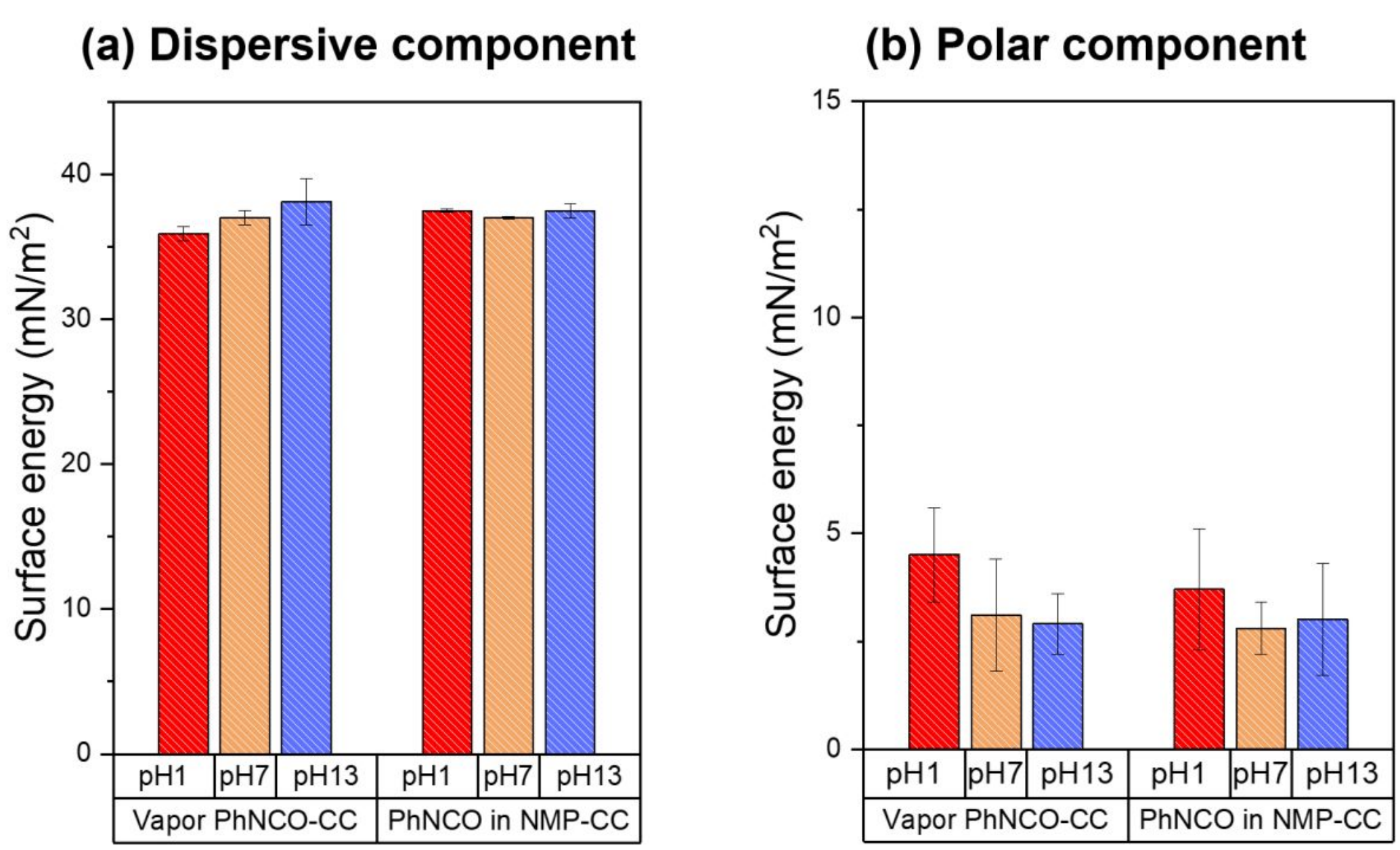

Figure S6. (a) Dispersive and (b) polar components of the surface energy of clearcoat (CC) films treated with aqueous solutions at pH 1, 7, and 13, followed by exposure to phenyl isocyanate vapor and by solution-phase reaction with phenyl isocyanate (Ph–NCO) for 10 min. Error bars represent the standard deviation from three independent measurements.

References

(1) Ryu, J.; Guo, Y.; Chen, S.; Choi, J.; Chen, X.; Kim, S. H. First-Principles Prediction of Vibrational Sum Frequency Generation Features of Phenyl Groups at the 2D Interface with C_∞ Symmetry. *Langmuir* **2025**, 41 (25), 16102-16114.

(2) Ryu, J.; Chen, S.; Choi, J.; Chen, X.; Kim, S. H. DFT-based calculation of vibrational sum frequency generation spectral features of crystalline β-sheets in silk: Polarization and azimuth angle dependences. *J. Chem. Phys.* **2024**, *161* (23), 234901.

(3) Choi, J.; Kwansa, A. L.; Yingling, Y. G.; Kim, S. H. DFT-Based Calculation of Molecular Hyperpolarizability and SFG Intensity of Symmetric and Asymmetric Stretch Modes of Alkyl Groups. *J. Phys. Chem. B* **2023**, 127 (39), 8456-8467.
